# Urgent-care antibiotic prescribing: An exploratory analysis to evaluate health inequities

**DOI:** 10.1017/ash.2022.329

**Published:** 2022-11-14

**Authors:** Allan M. Seibert, Adam L. Hersh, Payal K. Patel, Michelle Matheu, Valoree Stanfield, Nora Fino, Lauri A. Hicks, Sharon V. Tsay, Sarah Kabbani, Edward Stenehjem

**Affiliations:** 1 Division of Infectious Diseases and Clinical Epidemiology, Intermountain Healthcare, Murray, Utah; 2 Division of Pediatric Infectious Diseases, Department of Pediatrics, University of Utah, Salt Lake City, Utah; 3 Office of Research, Intermountain Healthcare, Salt Lake City, Utah; 4 Division of Epidemiology, Department of Internal Medicine, University of Utah, Salt Lake City, Utah; 5 Division of Healthcare Quality Promotion, Centers for Disease Control and Prevention, Atlanta, Georgia

## Abstract

Healthcare disparities and inequities exist in a variety of environments and manifest in diagnostic and therapeutic measures. In this commentary, we highlight our experience examining our organization’s urgent care respiratory encounter antibiotic prescribing practices. We identified differences in prescribing based on several individual characteristics including patient age, race, ethnicity, preferred language, and patient and/or clinician gender. Our approach can serve as an electronic health record (EHR)–based methodology for disparity and inequity audits in other systems and for other conditions.

Nearly 40% of urgent-care encounters may be associated with outpatient antibiotic prescriptions. Urgent-care centers are also associated with the highest rate of inappropriate prescribing for respiratory tract infections (RTIs) in the United States.^11,12^ Even though other conditions, such as genitourinary infections, may have objective elements to guide therapy decisions, RTI treatment may vary more between clinicians. Studies have also identified racial, ethnic, and geographic antibiotic prescribing disparities that may represent inequitable care.^13–20^ Urgent-care clinics have been a focus for antibiotic stewardship interventions across Intermountain Healthcare. As part of an organizational commitment to health equity, we conducted an exploratory, EHR-based analysis of antibiotic prescribing for respiratory encounters in our urgent-care centers to identify potential inequities in antibiotic prescribing.

## Healthcare disparities and inequities: Definitions for the current era

The Centers for Disease Control and Prevention (CDC) defines health equity as the state in which everyone has a fair and just opportunity to attain their highest level of health.^1^ A disparity is a difference in health, services, or outcomes by some variable (eg, age, race, or insurance), which may or may not be clinically justifiable. These variables have often been collected in the medical record for reasons unrelated to their influence on health measures. Contemporary definitions and descriptions for health equity, disparity, inequity, and determinants of health are presented in Table 1. Multiple interconnected determinants of health, structural racism, and provider and system biases contribute to disparities and inequities across the healthcare spectrum.^2–10^

**Table 1.** Contemporary Health Equity, Disparity, and Inequity Definitions

Health Equity	Health Disparity	Health Inequity	Determinants of Health
• Everyone has a fair and just opportunity to attain the highest level of health possible.	• Preventable differences in disease burden, injury, violence, services, outcomes, or any opportunity to achieve optimal health care by some variable (eg age, race, insurance), which may or may not be clinically justifiable • Often experienced by populations that have been disadvantaged by social or economic status, geographic location, sexuality, language, or environment • Racial and ethnic minorities, women, people in the Lesbian, gay, bisexual, transgender, queer, intersex, and others who do not identify as cisgender or heterosexual (LGBTQI+) community, those with limited English proficiency, and other populations experience health disparities	• Extends from the definition of health disparity • Any unjust disparity due to implicit or explicit biases at the individual or societal level, historical structures, social mechanisms, or other pressures	• Social: education, insurance • Economic: personal and generational wealth or poverty • Geographic/Environmental: urban, rural, greenspace, zip code, area deprivation index • Personal: sexual orientation, gender identity • Behavioral: diet, exercise • Biologic: comorbidities, age • Health systems: provider and system bias

Note. Use and meaning of these terms continues to evolve.^1–3^ Determinants of health encompass many, often interconnected, categories.

## A test case for evaluating health inequities: Antibiotic prescribing for respiratory conditions in urgent care

Intermountain Health is a nonprofit, integrated, healthcare delivery system in the Mountain West that operates 38 urgent-care clinics. We limited our exploratory analysis to encounters from July 1, 2018, to June 30, 2019, among adults aged ≥18 years. We selected patient characteristics for assessment based on data availability in our electronic health record (EHR). These included age group (18–64 years and ≥65 years), race, ethnicity, preferred language, clinician–patient sex combination, and clinician type (physician or advanced practice clinician [APC]). Patient race, ethnicity, preferred language, and sex were self-reported. Additionally, we included body mass index (BMI) ≥25 and <25 (overweight/obese and nonobese). Individual respiratory encounters were identified using a validated methodology based on *International Classification of Diseases, Tenth Edition* codes.^21^ Antibiotic prescribing rates for respiratory conditions overall and rates for tier 3 respiratory conditions (ie, conditions in which antibiotics are not indicated such as acute uncomplicated bronchitis) were assessed.^22^ Because no standard definition exists to identify a disparity or inequity, we considered an absolute percentage difference between groups within a characteristic group of ≥5.0% to represent a potential disparity or inequity for which further evaluation was merited.

We identified 122,930 (88.1%) respiratory urgent-care encounters in adults aged 18–65 years. Among them, 98% of patients were White, 89.6% were non-Hispanic, 98.3% preferred English, 60.4% were female, and 85.3% were seen by physicians. Of non-English speakers, Spanish was the preferred language (77.1%). Most clinicians were male (77.0%); 59,442 (42.6%) of all respiratory encounters were for tier 3 conditions; and 68.3% of encounters occurred in overweight adults (BMI ≥ 25).

Patient groups in which notable differences were identified included age, race, ethnicity, and preferred language (Table 2). Adults aged ≥65 years received more prescriptions for tier 3 conditions than patients aged 18–65 years (30.2% vs 20.8%). White patients received antibiotic prescriptions more frequently than their nonwhite counterparts overall (50.1% vs 39.5%) and for tier 3 encounters (22.8% vs 14.9%). Non-Hispanic patients received antibiotic prescriptions overall more frequently than Hispanic patients (50.0% vs 43.9%). Patients who preferred English received more overall antibiotic prescriptions compared to non-English preferred speakers (49.6% vs 43.2%). Among clinician groups, female clinicians prescribed antibiotics for tier 3 conditions less frequently than their male colleagues (18.5% vs 23.6%) and this distinction was preserved regardless of patient gender. APCs prescribed antibiotics overall and for tier 3 conditions similarly to physicians.

**Table 2.** Antibiotic Prescribing for Urgent-Care Respiratory Encounters in Patients Aged ≥18 Years Between July 1, 2018, and June 30, 2019

Characteristic	Overall Received Abx	Tier 3 Received Abx^a^
**Age group, no. (%)**		
18–64 y	60,741 (49.4)	**10,350 (20.8)**
≥65 y	8,322 (50.1)	**2,924 (30.2)**
**BMI, no. (%)**		
Nonobese (BMI <25)	17,234 (47.4)	2,961 (20.7)
Overweight & obese (BMI ≥25)	47,917 (50.3)	9,667 (23.2)
**White race, no. (%)**		
No	**1,017 (39.5)**	**189 (14.9)**
Yes	**64,697 (50.1)**	**12,402 (22.8)**
**Ethnicity, no. (%)**		
Hispanic	**5,311 (43.9)**	1,043 (18.9)
Non-Hispanic	**62,348 (50.0)**	11,990 (22.8)
**Patient preferred language, no. (%)**		
English	**68,025 (49.6)**	13,032 (22.4)
Non-English	**965 (43.2)**	241 (20.3)
**Patient female, no. (%)**		
No	27,346 (49.6)	5,875 (23.9)
Yes	41,713 (49.5)	7,398 (21.3)
**Clinician female, no. (%)**		
No	54,111 (50.3)	**10,705 (23.6)**
Yes	14,952 (46.7)	**2,569 (18.5)**
**Patient–clinician sex combination**		
Female clinician, female patient	9,127 (46.7)	1,470 (17.6)
Female clinician, male patient	5,824 (46.7)	**1,099 (19.6)**
Male clinician, male patient	21,522 (50.4)	**4,776 (25.2)**
Male clinician, female patient	32,586 (50.3)	5,928 (22.4)
**Clinician type, no. (%)**		
MD/DO	58,364 (49.0)	11,623 (22.8)
Advanced practice clinician	10,699 (52.3)	1,651 (19.5)

Note. Abx, antibiotics prescription; BMO, body mass index; MD/DO, medical doctor/doctor of osteopathy. Tier 3 codes are those where antibiotics are not indicated (eg, acute uncomplicated bronchitis).

a Absolute differences of ≥5.0% between groups within each category are indicated in bold.

## Results

Antibiotic prescribing practices varied by patient and clinician characteristics including age, race and ethnicity, sex, and language. Similar to prior studies, we observed White, non-Hispanic, and patients whose preferred language is English received antibiotic prescriptions at greater rates than non-White, Hispanic, and non-English speaking patients.^14,15,17,20^ Other studies have also described racial and age-related antibiotic prescribing differences prior to and during the pandemic.^23,24^ The reasons for these differences are unclear and merit further investigation. These findings could serve as a focus for future stewardship efforts to evaluate whether they relate to clinician or system biases, patient expectations, or some combination of these or other factors.

Female clinicians appeared less likely to prescribe antibiotics for tier 3 conditions regardless of patient sex. Prior studies have shown mixed results regarding the impact of clinician sex on guideline adherence in cardiology and outpatient antibiotic prescribing.^25–27^ Similar rates of antibiotic prescriptions for tier 3 conditions and overall prescribing between physicians and APCs is notable for its contrast with prior data suggesting higher rates of prescribing by APCs.^17^ This finding could reflect the Intermountain Healthcare urgent-care system involving all clinicians in education, tracking, and stewardship efforts. We did not identify differences ≥5.0% in overall or tier 3 antibiotic prescribing based on BMI; however, obesity has been shown to affect healthcare engagement, expenditures, and outcomes and should be included in inequity evaluations along with other chronic conditions and comorbidities.^28–34^

Our evaluation had several limitations. We have reported observed differences in a univariate analysis, and we did not assess for confounding. This study was an exploratory, EHR-based investigation, and we sought to identify areas in which potential inequities might exist. Further study and multivariable modeling could elucidate which features are most strongly associated with inequitable prescribing and could aid in identifying actionable areas for interventions. Small numbers of non-White, Hispanic, and non-English speaking patients may limit the generalizability of these findings. Limited sexual orientation and nonbinary gender identity (SOGI) data within our EHR precluded our ability to evaluate differences in care received by LGBTQI+ patients. Efforts are underway to improve capturing SOGI information along with other patient characteristics to optimize future inequity audits across our system. Lastly, our study encompassed the period prior to the COVID-19 pandemic.

## Future directions: Auditing (and resolving) disparities and inequities

Our EHR-based evaluation of health disparities and inequities in antibiotic prescribing can be used by health systems to examine other clinical conditions, therapeutics, and patient outcomes. Healthcare systems should create diverse teams of researchers, clinicians, and community members to evaluate observed differences and identify actions to achieve equity in healthcare delivery in the unique context in which any healthcare organization operates. Modification of system-level factors (eg, language support services, clinician language fluency, operational hours, telehealth accessibility, and a diverse system leadership team) should be considered in addition to evaluating and addressing clinician bias. This approach may also highlight and reinforce equitable care when observed. Although this exploratory analysis has limitations, the value of our approach using EHR data, and working to improve its collection and quality, lies in presenting a sustainable and adaptable mechanism to identify and monitor interventions that aim to reduce health inequities in healthcare delivery.
